# The Effect of the COVID-19 Pandemic on People with Parkinson’s Disease

**DOI:** 10.3233/JPD-202249

**Published:** 2020-10-27

**Authors:** Ethan G. Brown, Lana M. Chahine, Samuel M. Goldman, Monica Korell, Emerald Mann, Daniel R. Kinel, Vanessa Arnedo, Kenneth L. Marek, Caroline M. Tanner

**Affiliations:** a Department of Neurology, Weill Institute for the Neurosciences, University of California San Francisco, & San Francisco VA Health Care System, San Francisco, CA, USA; b Department of Neurology, University of Pittsburgh, Pittsburgh, PA, USA; c Department of Occupational and Environmental Medicine, University of California San Francisco, & San Francisco VA Health Care System, San Francisco, CA, USA; d University of Rochester, Rochester, NY, USA; e Michael J. Fox Foundation, New York, NY, USA; f The Institute for Neurodegenerative Disorders, New Haven, CT, USA

**Keywords:** COVID-19, Parkinson’s disease, social isolation, health care access, telemedicine

## Abstract

**Background:**

The effect of the COVID-19 pandemic on people with Parkinson’s disease (PD) is poorly understood.

**Objective:**

To rapidly identify areas of need and improve care in people with PD during the COVID-19 pandemic, we deployed a survey to assess COVID-19 symptoms and the pandemic’s effect among those with and without COVID-19.

**Methods:**

People with and without PD participating in the online study Fox Insight (FI) were invited to complete a survey between April 23 and May 23, 2020. Among people reporting COVID-19 diagnoses, we compared symptoms and outcomes in people with and without PD. Among people not reporting COVID-19, we assessed access to healthcare and services and PD symptoms.

**Results:**

7,209/9,762 active FI users responded (approximately 74% response rate), 5,429 people with PD and 1,452 without PD. COVID-19 diagnoses were reported by 51 people with and 26 without PD. Complications were more frequent in people with longer PD duration. People with PD and COVID-19 experienced new or worsening motor (63%) and nonmotor (75%) symptoms. People with PD not diagnosed with COVID-19 reported disrupted medical care (64%), exercise (21%), and social activities (57%), and worsened motor (43%) and non-motor (52%) symptoms. Disruptions were more common for those living alone, with lower income and non-White race.

**Conclusions:**

The COVID-19 pandemic is associated with wide-ranging effects on people with PD, and certain groups may be at particular risk. FI provides a rapid, patient-centered means to assess these effects and identify needs that can be used to improve the health of people with PD.

## INTRODUCTION

SARS-CoV-2 infection and the societal changes associated with the COVID-19 pandemic may have particularly severe consequences for people with chronic neurologic diseases, such as Parkinson’s disease (PD). Many people with PD are concerned about COVID-19 risk [1], and indeed early reports [2–4] describe worsening of parkinsonian symptoms during infection and poor outcomes in some cases. Among those not infected, social distancing guidelines may lead to social isolation, a known risk factor for poor outcomes [5], and reduced access to care [6], all of which are additional concerns of people with PD [1]. Rapid changes in healthcare delivery to people with PD have been developed, including recommendations on implementing telemedicine [7] and managing advanced therapies remotely [8, 9], but these resources are not universally available [10]. The persistent spread of the pandemic and the associated global disruption highlight the urgent need for increased understanding of its effect and mitigating strategies in people with PD.

We surveyed participants in Fox Insight (FI), an online study that involves thousands of people with and without PD [11], between April and May, 2020. Our study had several primary aims. First, we sought to understand the symptoms and outcomes of SARS CoV-2 infection in people with and without PD to determine how the disease may affect people with PD differently. Second, we wanted to determine the effects of COVID-19 on motor and non-motor symptoms related to PD. Third, we wanted to understand the effects of the pandemic and associated public health measures on people with and without PD, even if not directly infected with SARS-CoV-2. We were particularly interested in how demographic factors, including age, sex, and socioeconomic factors, related to disruptions from the pandemic.

## METHODS

### Description of fox insight

Fox Insight (FI) is a fully remote study platform [12]. Participants ≥18 years old with and without PD complete surveys at regular intervals and are periodically invited to complete additional surveys targeting particular topics [11].

### Survey design

This was a cross-sectional study. The COVID-19 survey was designed through an iterative process involving multidisciplinary providers and clinical researchers with active feedback on survey content and format from people with PD.

### Study population and survey deployment

All FI participants were invited by email on April 23, 2020, with follow-up reminders on May 1 and May 11 to participate in the COVID-19 survey. We estimated the number of active participants based on either (1) registration within the six months prior to survey launch or (2) initiation of an assessment within the six months. Because spread of the pandemic and related attitudes, guidelines, and practices have changed rapidly, we limited analyses to surveys completed between April 23 and May 23, 2020. We excluded respondents with missing diagnostic or demographic data in FI and those who reported a diagnosis of PD before age 25.

### Assessments

Participant information comes both from FI longitudinal study assessments and direct questions of the COVID-19 survey. From FI, we obtained demographic information that participants provide annually. For covariate analysis, older age was defined as more than 65 years at completion of the survey. Respondents of “non-White race” include those who did not identify as White and identified as another race (either African American, Asian, American Indian or Alaska Native, Native Hawaiian or Pacific Islander, according to the choices in FI). Participants with a household income below $50,000, representing roughly the lowest tertile of respondents, were classified as having lower income. We obtained information about prior olfactory function from the Nonmotor Symptoms Questionnaire (NMSQ), using surveys from more than 4 months prior to the COVID survey. Several PD-related genes may be pertinent to COVID-19 infection: LRRK2 mutations may play a role in immunomodulation [13], while APOE *ɛ*4 homozygosity has been associated with increased COVID-19 risk [14] and, along with GBA, may increase susceptibility to cognitive impairment in people with PD [15]. Genotype was therefore included for the subset of people with PD in FI who had those data available [11]. Information about PD diagnosis was obtained both from FI and from our current survey; respondents with conflicting answers were excluded.

All subsequent assessments of COVID-19 and the effect of the pandemic were obtained through the deployed survey. We considered people to have COVID-19 if they reported that a definite or probable COVID-19 diagnosis had been made by a medical professional. People reporting a history of diabetes, HIV or AIDS, recent chemotherapy, current treatment with oral steroids or other immune suppressants were classified as immunocompromised. To understand how the pandemic is affecting both infected and non-infected people with and without PD, the survey was divided into three sections, corresponding to our aims: (1) COVID-19 symptoms, diagnosis, testing, risk factors, and treatment; (2) change in PD-related symptoms; and (3) effect of shelter-in-place orders and social distancing practices on healthcare access, social interactions, and other essential activities (the full survey is available at https://foxden.michaeljfox.org).

### Definition of outcomes

For our first aim, we assessed COVID-19 related outcomes including development of pneumonia, need for supplemental oxygen, hospitalization, intensive care unit (ICU) admission or ventilator support. For our second aim, we evaluated changes in individual PD-related motor and non-motor symptoms by domain: motor problems (imbalance, falling, tremor, slow movement, stiffness, dysphagia, difficulty eating, increased OFF time, dyskinesia), cognitive problems (confusion, hallucinations, difficulty thinking, memory problems), sleep problems (acting out dreams, difficulty sleeping, excessive sleepiness, fatigue), mood symptoms (anxiety, depression, apathy), and autonomic problems (urinary problems, orthostasis, constipation). For our third aim, we assessed pandemic-associated disruption of medical care among people without COVID-19, daily essential activities, and exercise or social activities.

### Statistical analysis

Survey results and demographic information were summarized using descriptive statistics. We evaluated differences in means of continuous variables using Student’s *t*-test for normally distributed data and Wilcoxon rank sum for non-normally distributed. We used the chi-square test for comparison of categorical variables. For our main results, we constructed logistic regression models adjusting for age and sex. For COVID-19 related analyses, we performed sensitivity analyses restricting the cohort to only those reporting a definite COVID-19 diagnosis or a positive COVID-19 test. Participants who reported COVID-19 infection were excluded from analyses about the effects of social distancing to reduce confounding. All analyses were performed using R version 3.5.2 (12-20-2018). Statistical significance was defined as *p* < 0.05.

### Standard protocol approvals, registrations, and patient consents

The FI study and the survey for this COVID-19 study were approved by the New England IRB and informed consent was obtained online from all participants.

### Data availability policy

All survey questions and data are available at https://foxden.michaeljfox.org.

## RESULTS

### Respondent characteristics

**Fig. 1:**
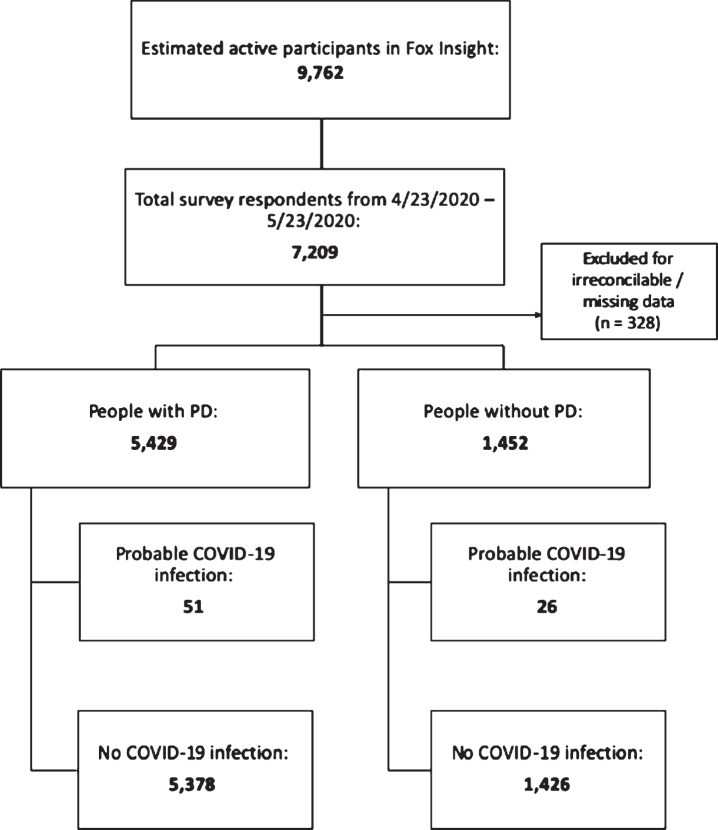
CONSORT diagram depicting number of respondents included in the study.

**Fig. 2:**
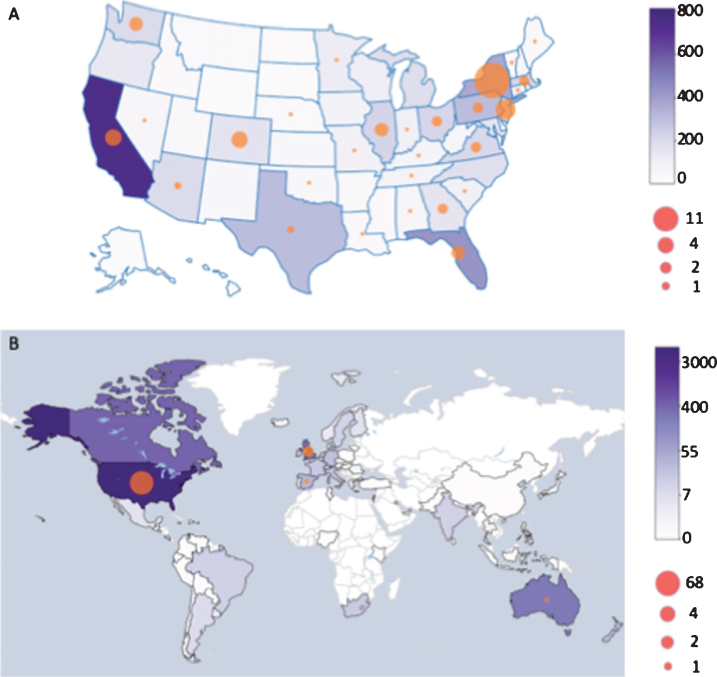
Distribution of survey responses and COVID-19 cases among respondents (A) within the US and (B) across the globe. The color of the state or country represents the number of survey responses and the size of the circles represents the number of COVID-19 diagnoses reported from each region.

As of May 23, 2020, 7,209 people with and without PD responded, representing 73.8% of the 9,762 Fox Insight participants active in the preceding 6 months. Three-hundred and twenty-eight people were excluded for irreconcilable or missing data, leaving 5,429 people with PD and 1,452 people without (Fig. 1). Responses were from all continents, but about 80% came from the United States (Fig. 2). Respondents with PD were older, more often male, had a higher prevalence of heart disease, a lower prevalence of immunocompromising conditions and lung disease, and had lower household income than people without PD (Table 1).

**Table 1:** Characteristics of respondents. Numbers in parentheses represent range for continuous variables and percentage of specific cohort for categorical variables. Immunocompromised defined as having a history of diabetes, HIV or AIDS, chemotherapy within the past year, taking steroid medications by mouth, or taking other immune suppressing medication. Note: genotype was not available for people without PD

	COVID-19 Infection		No COVID-19 Infection		
	PD N = 51	Not PD N = 26	*p*-value*	PD N = 5378	Not PD N = 1426	*p*-value
Age, mean in years (range)	65 (40–89)	57 (30–73)	**0.004**	68 (33–95)	61(19–94)	**<0.001**
Female, N (%)	27 (53)	24 (92)	**0.001**	2598 (48)	1115 (78)	**<0.001**
PD Duration, N (%)
0–3 y	21 (41)	––	1628 (30)	––
3–6 y	12 (24)	––	1649 (31)	––
6–9 y	9 (18)	––	981 (18)	––
>9 y	9 (18)	––	1114 (21)	––
Comorbidities, N(%)
Immunocompromised	11 (22)	8 (31)	0.5	637 (12)	198 (14)	**0.041**
Current Smoker	3 (5.9)	0 (0)	0.5	84 (1.6)	32 (2.2)	0.10
Former Smoker	20 (39)	7 (27)	0.4	1408 (26)	368 (26)	0.8
Heart Disease	10 (20)	1 (3.8)	0.087	440 (8.2)	90 (6.3)	**0.022**
Hypertension	13 (25)	8 (31)	0.8	1649 (31)	439 (31)	>0.9
Lung disease	7 (14)	11 (42)	**0.012**	436 (8.1)	198 (14)	**<0.001**
White, N (%)	51 (100)	26 (100)	5247 (98)	1396 (98)	0.5
Latinx, N (%)	4 (8.0)	0 (0)	0.3	168 (3.2)	42 (3.0)	0.7
Income< $50,000, N (%)	13 (28)	6 (25)	>0.9	1201 (26)	274 (22)	**0.002**
Lives Alone, N (%)	8 (19)	4 (17)	>0.9	630 (13)	256 (19)	**<0.001**
Genotype, N (% with genotype data)
APOE *ɛ*4 allele (*n* = 599)
None	3 (100)	––	450 (76)	––
One	0	––	135 (23)	––
Two	0	––	7 (1.2)	––
GBA Mutation (*n* = 609)	0	––	39 (6.5)	––
LRRK2 G2019S (*n* = 640)	0	––	28 (4.4)	––

*p-values represent comparisons between people with and without PD.

### COVID-19 symptoms and outcomes in people with PD

COVID-19 diagnoses were reported by 51 people with PD (22 definite, 29 probable) and 26 people without PD (7 definite, 19 probable). Positive tests were reported in 17 people with PD and 6 without PD. One person with PD reported a positive COVID-19 test but was asymptomatic and was not included in analyses of COVID-19-associated disease features. Among people with PD, those with COVID-19 were more likely to smoke (5.9% vs 1.6%, *p* = 0.048) and have a history of heart disease (20% vs 8.2%, *p* = 0.008). Compared to people without PD who had COVID-19, people with PD who had COVID-19 were more likely to be older, male, and less likely to have lung disease (Table 1).

Behavioral and environmental risk factors for COVID-19 were more common in people without PD than people with PD: 100% of people without PD with COVID-19 reported a professional or recreational activity that potentially put them at risk, such as travel or having an occupation that could have increased their exposure, whereas only 75% of people with PD with COVID-19 could identify any such activity (*p* = 0.012). In particular, people without PD with COVID-19 more often reported working in healthcare, as a first responder, at an adult or childcare facility, or at another essential job (46% vs 14%, *p* = 0.004). People with PD and COVID-19 were more likely to have a household or other personal contact that was diagnosed with, suspected to have, or had symptoms consistent with COVID-19 compared to people with PD without COVID-19 (59% vs 7.7%, *p* < 0.001). The types of COVID-19 symptoms were generally similar in people with PD and those without except for more frequent chills and pulmonary symptoms in people without PD (Table 2). Information about prior olfactory function was available in 37 people with PD, 24 of whom denied prior loss of sense of smell, and 23 people without PD, 20 of whom denied prior loss of sense of smell. In the setting of COVID, the new onset of hyposmia was reported by 14 (38%) people with PD and 9 (39%) of people without PD, and worsening of existing hyposmia was reported by 4 (11%) of people with PD and none of those without PD. Among people with COVID-19, there were slight differences in several reported outcomes between those with and without PD, but none were statistically significant (Table 2). Longer PD-duration was associated with a higher risk of pneumonia, the need for supplemental oxygen, or hospitalization (44% among people with PD for greater than 9 years vs 14% among people with PD with equal to or less than 9 years, aOR 5.44, 95% CI 1.04–30.5, *p* = 0.043).

**Table 2:** Symptoms and outcomes of COVID-19 in people with and without PD. *P*-value represents results of chi-square test. Upper respiratory symptoms include congestion and sore throat. Lower respiratory symptoms include chest tightness, chest pain, and shortness of breath. GI symptoms include nausea, vomiting, diarrhea, and stomach pain. O2, oxygen; ICU, intensive care unit

COVID-19 Symptoms and Outcomes in People with and without PD			
	Symptoms			
	PD, N = 51	Not PD, N = 26	*p*-value
Fever	32 (63%)	20 (77%)	0.3
Chills	28 (55%)	21 (81%)	**0.048**
Cough	36 (71%)	25 (96%)	**0.020**
Upper Respiratory	35 (69%)	20 (77%)	0.6
Lower Respiratory	29 (57%)	24 (92%)	**0.004**
GI	34 (67%)	20 (77%)	0.5
Muscle/Joint Pain	39 (76%)	24 (92%)	0.2
Sleepiness	44 (86%)	26 (100%)	0.12
Lightheadedness	34 (67%)	23 (88%)	0.074
	Outcomes			
Pneumonia	4 (7.8%)	3 (12%)	>0.9
Supplemental O2	6 (12%)	2 (7.7%)	0.9
Hospitalized	5 (9.8%)	2 (7.7%)	>0.9
ICU	2 (3.9%)	1 (2.0%)	>0.9
Ventilator	1 (2.0%)	0 (0%)	>0.9

### The effects of COVID-19 on PD-related symptoms

During COVID-19 infection, people with PD reported worsening of many PD-related symptoms (Fig. 2). New motor symptoms were reported by 18%, and 55% reported worsening of at least one existing motor symptom. New and worsening non-motor symptoms were reported for all domains: mood (20% new, 51% worsening), cognition (7.8% new, 41% worsening), sleep (12% new, 59% worsening), and autonomic (7.8% new, 29% worsening).

### The effects of the COVID-19 pandemic among people with and without PD

Among those without COVID-19, healthcare was altered due to the pandemic in 62% of all respondents, including cancelled appointments, reduced in-home care or difficulty obtaining medications. Only cancellations in rehabilitation services were more common in people with PD (17% people with PD vs 5.5% not PD, *p* < 0.001). Among people with PD, disruption in medical care was more likely in those with longer PD duration (41% PD duration >9 years vs. 32% for PD duration 0–3 years, adjusted odds ratio (aOR) = 1.47, 95% CI 1.26–1.73, *p* < 0.001) (Table 3). Race and lower income were independently associated with difficulty obtaining PD medications (non-White race 13% vs 7.3%, aOR 1.98 95% CI 1.05–3.45, *p* = 0.023, and lower income 10% vs 7.2%, aOR 1.36, 95% CI 1.07–1.72, *p* = 0.01); arguably more people of Latinx ethnicity reported difficulty obtaining medications (14% vs 7.5%, 1.61, 95% CI 0.93–2.63, *p* = 0.07). Telemedicine appointments were reported by 39% of people with PD, but those with lower household income were less likely to attend healthcare appointments through telemedicine (40% vs 35%, aOR 0.79, 95% CI 0.69, 0.90, *p* = <0.001).

**Table 3:** Interruptions in PD-related medical care stratified by disease duration among people with PD without COVID-19 infection

Disruptions of Medical Care in People with PD Without COVID-19 Related to the COVID-19 Pandemic					
PD Duration (y)	0–3 N = 1628	3–6 N = 1649	6–9 N = 981	>9 N = 1114	*p*-value*^2^*
Cancelled or postponed rehab therapy	396 (24%)	426 (26%)	265 (27%)	331 (30%)	**0.016**
Have lost or reduced in-home care services	35 (2.1%)	47 (2.9%)	35 (3.6%)	69 (6.2%)	**<0.001**
Had to cancel healthcare appts	771 (47%)	813 (49%)	511 (52%)	593 (53%)	**0.011**
Cancelled or postponed mental health care	78 (4.8%)	68 (4.1%)	28 (2.9%)	48 (4.3%)	0.12
Problems obtaining meds for PD	118 (7.2%)	114 (6.9%)	83 (8.5%)	96 (8.6%)	0.3
Cancelled or postponed Botox Treatment	21 (1.3%)	47 (2.9%)	25 (2.5%)	45 (4.0%)	**<0.001**
Cancelled or postponed DBS Surgery	7 (0.4%)	10 (0.6%)	16 (1.6%)	14 (1.3%)	**0.004**
Cancelled or postponed DBS Battery Replacement	0 (0%)	1 (<0.1%)	3 (0.3%)	7 (0.6%)	**0.002**
Cancelled or postponed DBS programming	3 (0.2%)	14 (0.8%)	22 (2.2%)	54 (4.8%)	**<0.001**

At least one essential daily activity was disrupted in 35% of people with PD. Essential daily activities were more often disrupted in people with PD who live alone compared to other people with PD, including getting food (12% vs 8.7%, aOR 1.50, 95% CI 1.14–1.94, *p* = 0.003) and getting regular homecare / housekeeping 15% vs 9.0%, aOR 1.50, 95% 1.16–1.91, *p* = 0.001). Other regular behaviors disrupted due to the COVID-19 pandemic included cancelled exercise (21% people with PD) and social activities (57%), although many people found alternative ways, such as online classes, to continue these activities (Table 4). people with PD with lower income were less likely to report alternative means of exercising (32% vs 44%, aOR 0.57, 0.50–0.66, *p* < 0.001) or social activities (49% vs 58%, aOR 0.66, 0.58–0.76, *p* < 0.001); older people with PD were less likely to use alternative ways to exercise (39% vs 44%, aOR 0.82 (95% CI 0.73, 0.93), *p* = 0.002).

**Table 4:** Interruptions in activities during the COVID-19 pandemic among people with PD but not infected by COVID-19

Changes in activities among people with PD not infected by COVID				
	Cancelled	Postponed	Conducted via alternative method(s)	Not applicable
Exercise	1140 (21%)	427 (7.9%)	2187 (41%)	1624 (30%)
Seeing Family	1316 (24%)	1175 (22%)	2062 (38%)	825 (15%)
Seeing Friends	1572 (29%)	1341 (25%)	1925 (36%)	540 (10%)
Support Group Attendance	893 (17%)	240 (4.5%)	529 (9.8%)	3716 (69%)
Volunteer Activities	1062 (20%)	459 (8.5%)	423 (7.9%)	3434 (64%)
Religious Activities	1095 (20%)	390 (7.3%)	1266 (24%)	2627 (49%)
Community Activities	2156 (40%)	940 (17%)	620 (12%)	1662 (31%)

The pandemic was also disruptive to research participation among the cohort. Among the 16% of people with PD without COVID-19 who had been participating in research, 40% had to cancel and 35% had to postpone in-person research visits, while 25% were able to conduct research visits through alternative methods. Overall, 6.3% of people with PD felt that the COVID-19 pandemic had made them more likely to participate in research, while 11% felt that the pandemic had made them less likely to participate, and 83% reported no change in their likelihood to participate in research.

**Fig. 3:**
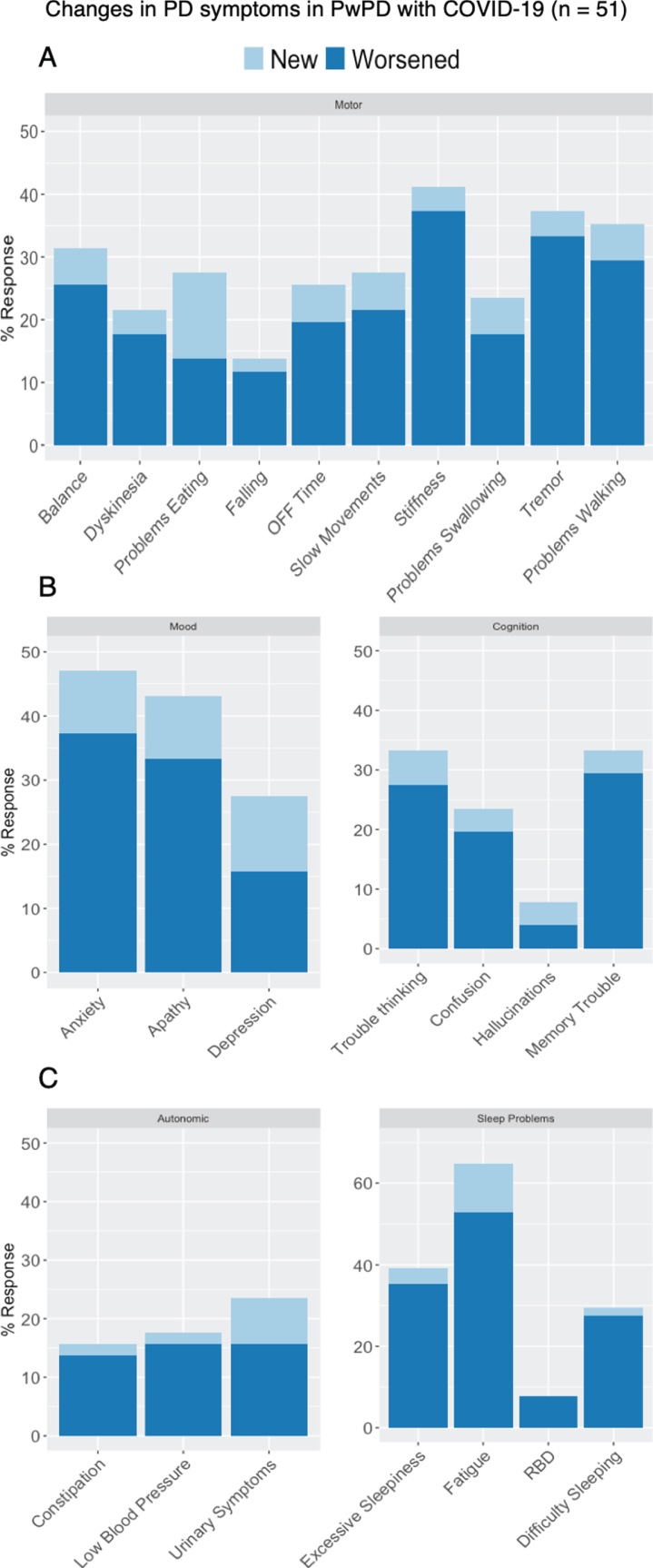
Changes in (A) motor and (B - C) non-motor PD-related symptoms among respondents with PD and COVID-19 (*n* = 51).

Among people with PD who did not have COVID-19, new PD symptoms emerged and existing symptoms worsened in all major domains (motor: 6.2% new, 41% worsened; mood: 6.5 % new, 30 % worsened; cognitive: 2.5 % new, 16 % worsened; sleep: 4.5% new, 32% worsened; autonomic: 2.6% new, 18% worsened (Fig. 3). Respondents who experienced interruptions to PD-related medical care were also more likely to experience new or worsening symptoms in all domains (motor symptoms: 56% vs 36%, aOR 2.21, 95% CI 1.97–2.48, *p* < 0.001; cognitive problems: 24% vs 14%, aOR 1.92, 95% CI 1.66–2.22, *p* < 0.001; mood symptoms: 42% vs 30%, aOR 1.70, 95% CI 1.51–1.92, *p* < 0.001; autonomic symptoms: 27% vs 17%, aOR 1.75, 95% CI 1.53–2.00, *p* < 0.001; and sleep problems: 42% vs 31%, aOR 1.60, 95% CI 1.42–1.79 *p* < 0.001). New onset motor symptoms in particular were more likely in those that had disruption of medical care (8.2% vs 5.1% aOR 1.63, 95% CI 1.31–2.04, *p* < 0.001). Respondents who experienced interruptions to exercise, social activities or were asked to self-isolate were also more likely to report worsening of PD symptoms (Table 5). Neither the number of APOE *ɛ*4 alleles nor PD-associated mutations in LRRK2 were associated with an increased risk of being diagnosed with COVID-19, and the number of APOE *ɛ*4 alleles and GBA mutations were not associated with new or worsening PD-related symptoms during the pandemic.

**Fig. 4:**
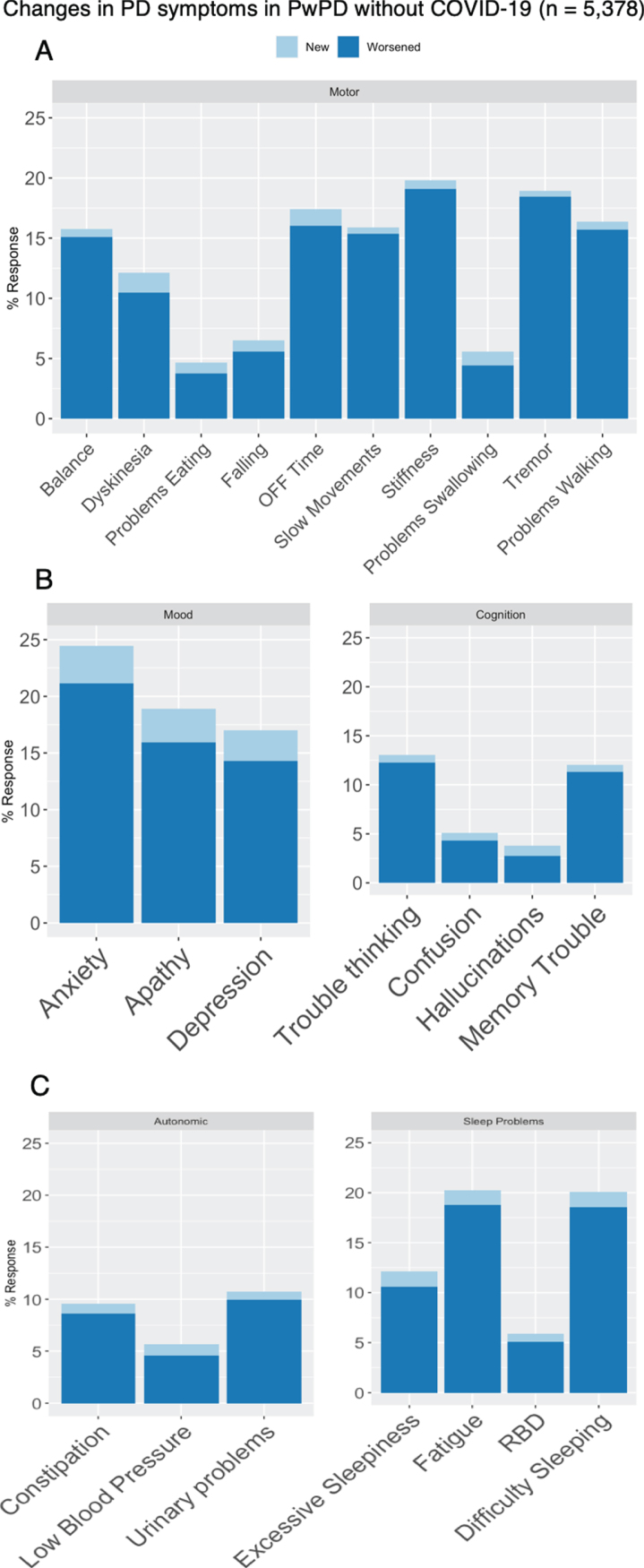
Changes in (A) motor and (B - C) non-motor PD-related symptoms among respondents with PD without COVID-19 (*n* = 5,378).

**Table 5:** Risk of new or worsening PD-related symptoms in various domains after cancelled or postponed exercise, cancelled or postponed social activities, or being asked to self-isolate/quarantine during the COVID-19 pandemic among people with PD and without COVID-19. PD-symptom domains include motor (problems with walking, balance, falling, tremor, slow movements, stiffness, swallowing, eating, increased off-time, or dyskinesia), cognition (problems with thinking, memory, confusion, hallucinations), mood (anxiety, depression, apathy), autonomic (constipation, urinary problems, low blood pressure), and sleep (insomnia, fatigue, excessive sleepiness, REM sleep behavior disorder). Odds ratios are shown with 95% confidence intervals in parentheses. Odds ratios are adjusted for the other factors presented. *p < 0.01, **p < 0.001

Factors Associated with Worsening PD-related Symptoms during COVID-19					
Pandemic among People with PD without COVID-19					
	Motor	Cognitive	Mood	Autonomic	Sleep
Exercise Activities	1.31**	1.28*	1.21*	1.23*	1.34**
Cancelled/Postponed	(1.16, 1.49)	(1.10, 1.50)	(1.07, 1.38)	(1.06, 1.42)	(1.18, 1.52)
Social Activities	1.69**	1.41**	1.63**	1.46**	1.31**
Cancelled/Postponed	(1.47, 1.95)	(1.17, 1.72)	(1.40, 1.90)	(1.22, 1.76)	(1.13, 1.51)
Asked to self-	1.78**	1.90**	1.69**	1.81**	1.71**
isolate/quarantine	(1.49, 2.13)	(1.55, 2.32)	(1.41, 2.02)	(1.48, 2.19)	(1.43, 2.04)

Women with COVID-19 more often reported new or worsening cognitive symptoms (67% women vs 25% men, aOR 6.00, 95% CI 1.82–22.3, *p* = 0.005). New or worsening symptoms were also more common in women with PD but without COVID-19: motor symptoms (49% women vs 38% men, aOR 1.63, 95% CI 1.46–1.81. *p* < 0.001), mood symptoms (42% women vs 27% men, aOR 1.89, 95% CI 1.69–2.12, *p* < 0.001), sleep problems (39% women vs 31% men, aOR 1.43, 95% CI 1.28–1.60, *p* < 0.001), and autonomic problems (23% women vs 18% men, aOR 1.33, 95% CI 1.17–1.52, *p* < 0.001). In analyses adjusting for social factors more commonly experienced by women with PD (living alone, disrupted home care, interruptions in medical care), women with and without COVID-19 were still at greater risk of symptom worsening.

## DISCUSSION

This large, participant reported study demonstrates the profound effect COVID-19 has had on individuals with PD. Thousands of people completed the survey in just a few weeks, illustrating the value of online research in providing rapid responses from an engaged community despite limits to in-person interactions.

### Occurrence of COVID-19 in people with and without PD

Overall, roughly 1% of our respondents with PD reported a definite or probable diagnosis of COVID-19. Most of the respondents were from the U.S.A., where the cumulative cases was around 500 per 100,000 by May 23, 2020 (https://coronavirus.jhu.edu/data/cumulative-cases). The higher proportion of COVID-19 cases in our population may indicate that people who were infected were more motivated to complete our survey. Importantly, our study was not designed for comparing population rates of people with and without PD, since people with PD are over-represented in FI, and we could only capture people able to complete an online survey.

A recent report suggested that COVID-19 infection is more common in people homozygous for APOE *ɛ*4 [14]. Among the people with PD with genetic testing available, no one with COVID-19 had an APOE *ɛ*4 allele. Furthermore, PD-related mutations were not related to the risk of COVID-19 or symptomatic worsening during the pandemic. However, as the prevalence of these mutations is low, our sample size may not have been big enough to detect a difference in associated risk.

### COVID-19 sequelae in people with and without PD

Among those with COVID-19, outcomes were largely similar between people with and without PD. Longer PD-duration was associated with a higher risk for poor outcomes. Similarly, a series of 10 cases identified through hospitals in Padua, Italy, and London, UK, suggested that people with PD of longer duration may be at risk of more severe illness and mortality from COVID-19 [2]. Preliminary reports from the CDC indicate that a high proportion of people with COVID-19 and neurologic disorders require hospitalization [16], and severity of other neurologic conditions has been associated with complications of COVID-19 [17]. Although the number of people with PD and COVID-19 studied is small, consistency among reports suggests that complications may be more frequent in advanced people with PD.

### PD-related symptoms and COVID-19

Both motor and non-motor symptoms of PD worsened during SARS-CoV-2 infection, including stiffness, tremor, difficulty walking, mood symptoms, cognition, and fatigue. Others have similarly reported worsening of PD-related symptoms either as presenting signs of COVID-19 [4] or during the course of the infection [2, 3]. Worsening of PD symptoms is commonly experienced in people with PD who have infectious illnesses [18], possibly due to systemic inflammation, altered dopaminergic signaling, or changes in medication absorption or pharmacokinetics [19]. Worsening of symptoms due to a direct infection of the CNS by SARS-CoV-2 is less likely. Although COVID-19 has been associated with changes on neuroimaging [20, 21] and SARS-CoV-2 RNA has been detected in cerebrospinal fluid [22, 23], a recent autopsy series of patients that died with COVID-19—all of whom experienced altered mental status—did not find immunohistochemical evidence of encephalitis or viral invasion into brain tissue [24]. Exacerbation of PD symptoms during COVID-19 may in part be due to the inflammatory reaction characteristic of the disease [25]. The consistent reporting of symptom exacerbation in people with PD due to COVID-19 emphasizes the need to consider COVID-19 as a possible explanation for suddenly worsening PD-related symptoms.

Hyposmia has been well described in people with COVID-19 [26]. Respondents to this survey with and without PD who reported COVID-19 also reported new onset hyposmia, at a similar rate to subjective reports in previous studies [26]. A small proportion of people with PD who already had hyposmia also reported worsening of this symptom.

A greater proportion of women than men with PD reported COVID-19. Other case series of people with PD and COVID-19 have not shown an excess of women [2, 3], though our study did capture more people. Women are relatively over-represented in the FI population [28] and may be more likely to respond to surveys, but this tendency should be equally distributed in those with and without COVID-19. Men with COVID-19 have been reported to have more severe disease than women [29, 30], while women may be more susceptible [31]. Women with COVID-19 may be over-represented in our study because they had relatively milder disease, and were therefore more able to answer surveys than men. Women with PD without COVID-19 reported worsening or new onset of PD symptoms more often than men without COVID-19 in all domains except cognition. Other studies have similarly suggested that women with PD report more mood symptoms [32, 33] and fewer cognitive problems [34] than men, and our findings may be more reflective of differences in symptoms between men and women with PD rather than reflecting changes due to the pandemic.

### Disruptions in PD-related care due to the COVID-19 pandemic

The vast majority of people with PD did not have COVID-19, yet most reported significant disruptions in many aspects of their daily lives due to the pandemic and consequent public health precautions. Longer disease duration and living alone increased the risk of disruption to medical care or other essential activities, indicating that these groups may need specific attention. Non-White race and lower household income were independently associated with difficulty obtaining medications, a particularly concerning finding that highlights the presence of barriers to healthcare access even among an online research cohort, and that these barriers are exacerbated during the pandemic. People with lower income were also less likely to find alternative means to conduct exercise and social activities and were less likely to use telemedicine. Among those with PD, lower socioeconomic status may already be associated with poor outcomes such as earlier exit from the work force [35] and higher mortality risk [36]. The rapid transformation of healthcare delivery increases access for many through telemedicine alternatives and will hopefully be a long-lasting consequence of the COVID-19 pandemic [7], but access for some already disenfranchised groups may be further reduced unless we are particularly conscious of this risk [37]. The finding that 35% of people with lower socioeconomic status were able to use telemedicine resources is encouraging that this service can be further expanded.

Interruption of medical care, exercise, and social activities were also associated with exacerbation of PD symptoms. The effect was even greater among those asked to socially isolate or quarantine. In the elderly, social isolation has been associated with more rapid cognitive decline [38], and quarantine in particular can lead to lasting psychological effects [39]. Individuals with PD are at high risk for anxiety [40], and may be particularly vulnerable to external stressors [41]. Developing ways to minimize these adverse consequences while maintaining safety precautions are needed.

### Limitations

Several limitations of this study must be acknowledged. Our response rate was moderate at 74% of active FI users, but still above the goal suggested for survey-based research [42]. Online surveys often have a lower response rate than in-person research [43], and many people may have had difficulty finding time to respond during the pandemic. We may have increased our response rate by waiting for longer than one month and sending more reminders, but we elected to analyze responses after one month, because we thought that the rapidly changing context of the pandemic would have made the survey more challenging to interpret had the window for responses been longer. The survey remains open and future analysis will evaluate whether or not subject response changes over time. Another limitation includes our reliance on self-report of COVID-19 infection and outcomes. In sensitivity analyses, applying more stringent criteria for COVID-19 classification of cases did not change our findings. Survey completion was naturally limited to people who were healthy enough to log-in online and fill out a survey, and we likely did not capture those with very advanced PD nor those with severe COVID-19 illness. Furthermore, we could not capture any cases of COVID-19 that resulted in death. People with PD and COVID-19 may have been less likely to fill out a survey, preventing our study from identifying true differences in infection risk or outcome severity. Additionally, certain populations were underrepresented, and the fact that we did see significantly greater disruption from the pandemic in some of these groups (e.g. non-White race, lower income) indicates that the true differences may be much larger. Finally while PD-diagnosis is self-reported in FI, available data indicate high accuracy of self-reported PD in research settings; in one study that verified self-reported diagnosis of PD through use of a Webcam, a neurologist agreed with the diagnosis in 97% of cases [44]. An effort to validate diagnoses in FI is currently underway.

### Conclusion and future directions

Our study raises important questions for future analyses. Anosmia in COVID-19, though usually transient [45], may represent true viral invasion of the olfactory bulbs [46]. Hyposmia predicts PD-associated clinical and pathological changes [47, 48]. This association, among other neurologic manifestations in people with COVID-19 [49, 50], has prompted worries about the possibility of SARS-CoV-2 infection triggering long-term neurodegeneration [51, 52], as was observed following the 1917-1918 pandemic [53]. This survey will provide useful baseline information for follow-up of respondents with COVID-19, to see if parkinsonism or other neurodegenerative diseases develop in those without PD, or if a different clinical course occurs in those with PD. Our survey population also provides the opportunity to investigate the long-term effects of COVID-19 on PD progression and of the social and public health aspects of the pandemic on people with PD. People with PD rely heavily on a network of outpatient healthcare, ancillary services, and social support, and interruptions of those services may have lasting implications that should be characterized longitudinally. FI has the capability to reach a broader and more diverse group than traditional, center-based research, and targeted recruitment will allow future studies to clarify the extent of and solutions for disparities in healthcare access during and after the pandemic.

In conclusion, out of about 5,000 people with PD, about 1% had a definite or probable COVID-19 diagnosis, and 75% of those infected experienced new or worsening PD-related symptoms. For people with PD without COVID-19, disruptions were frequent in PD-related medical care (64%), essential daily activities (35%), exercise (21%), and social activity (57%), and contributed to worsening of motor and non-motor symptoms especially in specific at-risk groups. Compensatory activities such as telemedicine and online exercise and social programs are important, though these are not available to everyone. As the pandemic and social distancing guidelines will likely last for some time, targeted strategies should be developed to provide support to patients with all levels of resources. The COVID-19 pandemic will likely have long-term repercussions; intervention to mitigate those effects in our patients should begin as soon as possible.

## CONFLICT OF INTEREST

E. G. Brown receives research support from the Michael J. Fox Foundation and the Gateway Institute for Brain Research Inc. He has received research support from Biogen Inc within the last two years. He has received honorarium from NEJM Knowledge+ as the Neurology Section Editor, and has received consulting fees from Oscar Health and Rune Labs Inc within the last two years.

L. M. Chahine receives research support from the Michael J Fox Foundation, UPMC Competitive Medical Research Fund, and University of Pittsburgh, is study site investigator for a study sponsored by Biogen, receives research support from the National Institutes of Health, receives royalties from Elsevier (for authorship), and receives royalties from Wolters Kluwel (for authorship).

S. M. Goldman’s research is supported by grants from the Michael J. Fox Foundation, VA CSR&D Merit, ATSDR/CDC, NIOSH, Department of Defense, Biogen Inc, UCSF Academic Senate.

M. Korell receives research support from the Michael J. Fox Foundation.

E. Mann reports nothing to disclose.

D. Kinel reports nothing to disclose.

V. Arnedo is an employee of The Michael J. Fox Foundation for Parkinson’s Research.

K. Marek reports grants from the Michael J Fox Foundation, Dept of Defense and personal fees from GE Healthcare, Roche, Neuropore, Proclara, UCB, Lysosomal Therapeutics, Inc, Neuroderm, Denali, Takeda, Samumed, Cerapsir, HANDL, Samus, Biohaven, Neuron23, Aprinoia, Genentech, Invicro.

C. M. Tanner reports grants from Parkinson Foundation, Gateway LLC, Roche/Genentech, Parkinson Study Group, Michael J Fox Foundation, NIH/NIA, NIH/NINDS, VA Merit, Dept of Defense, Biogen Idec, Roche Genentech and personal fees from Biogen Idec, Acorda, Adamas Therapeutics, Amneal, CNS Ratings, Grey Matter LLC, Northwestern University, Partners, Harvard U, Guidemark Health, Acadia, Neurocrine, Lundbeck and Cadent.
